# Feeling wronged: maternity care experiences of black, Sub-Saharan African migrant women living in Belgium during COVID-19

**DOI:** 10.3389/fgwh.2026.1692945

**Published:** 2026-03-23

**Authors:** Miranda Atembeshu, Dirk Lafaut

**Affiliations:** 1 Department of Political Science, Vrije Universiteit Brussel, Brussels, Belgium; 2 Ecole de Santé Publique - POLISSI, Université Libre de Bruxelles, Brussels, Belgium

**Keywords:** Critical Race Theory, migrant health, obstetric violence, pandemic, participant observation

## Abstract

**Introduction:**

This article examines the maternity care experiences of Black Sub-Saharan African migrant women living in Belgium during the COVID-19 pandemic, using the lens of Critical Race Theory (CRT). It explores how women describe their interactions with maternal healthcare providers and how intersecting factors-race, migration status, language, and gender - shaped their maternity care.

**Methods:**

The study employed a qualitative, narrative design combining semi-structured, in-depth interviews with 30 women who gave birth in Belgium between 2020 and 2022, alongside participant observation at a Born House. The data were analyzed through a multi-phase thematic analysis that, in line with CRT, focused on counter-stories.

**Results:**

Findings show that the pandemic and the measures to contain it compounded and exacerbated experiences of stereotyping, linguistic racism, and obstetric violence among Black migrant women. Stereotyping contributed to inadequate pain management, delayed diagnoses, and a lack of emotional support. COVID-19 measures - such as mask mandates, teleconsultations, and reduced availability of interpreters - intensified existing language barriers in interaction with maternal healthcare providers, enabling the expression of racist attitudes and reinforcing systemic exclusion from pregnancy-related decision-making. As a result, many women reported feeling wronged or coerced during and after childbirth. Mechanisms of redress did not accompany processes of blame attribution.

**Discussion:**

From a CRT perspective, we argue that pandemic measures without consideration of the specific needs of racialized migrants and linguistic minorities constitute a form of institutional neglect, reinforcing pre-existing inequities in maternity care. Tackling structural racism is not only a long-term imperative, but also requires acute awareness of the needs of racialized minorities when introducing new, ostensibly universal public health measures in times of crisis. View less

## Background

Social inequality in maternity care is a critical public health concern. Studies across Europe and North America consistently show that racialized migrants and women of color experience poorer maternal health outcomes and lower-quality care compared to native-born and white populations (1). A substantial body of research has documented that migrant women in Europe are at higher risk of adverse perinatal outcomes, including higher rates of preterm birth, low birth weight, and maternal mortality (2). These disparities are often linked to delayed access to antenatal care, inadequate communication, lack of culturally appropriate services, and lower socioeconomic status (3).

A systematic review showed that both maternal and fetal outcomes worsened globally during the COVID-19 pandemic, with an increase in maternal deaths, stillbirths, ruptured ectopic pregnancies, and maternal depression (4). More broadly, the recent COVID-19 pandemic contributed to important health disparities among racial/ethnic groups. Research - primarily conducted in the US and corroborated by studies in other Western countries - has shown that Black and Hispanic populations experienced disproportionately higher rates of COVID-19 infection, hospitalization, and COVID-19–related mortality compared to non-Hispanic White populations (5, 6). Divergent perspectives have been offered to explain these disparities in health outcomes. Ethnic and racial inequality in COVID-19-related deaths may be partially attributed to adverse social determinants of health, such as economic status and level of education (7) and by unequal healthcare access. However, structural racism and differences in workplace exposure also play a significant role (8, 9). Rather than higher case-fatality rates, increased exposure, especially among essential workers, who are disproportionately people of color, helps explain the disparities, as physical distancing policies often did not apply to these frontline occupations.

Since both COVID-19 and maternal mortality disproportionately affect racialized minorities, studying their simultaneous impact is highly valuable to understand how different structural mechanisms of exclusion intersect. Maternal mortality rates in the U.S. rose by 33% after the onset of the pandemic, disproportionately affecting non-white, Hispanic, and Black mothers, including migrant women (10). Scholars have argued that direct viral infection is not the only, nor the primary, way that the COVID-19 pandemic affected maternal mortality (11). An important debate centers on whether the adverse maternal outcomes have their genesis upstream by exacerbating factors negatively impacting the social determinants of health that already disfavor minority women, or downstream by the reduced quality of the healthcare system, e.g., by reducing the number of prenatal visits (11).

In response to the COVID-19 pandemic, countries worldwide implemented policies such as lockdowns and social distancing measures. Many healthcare institutions also developed a hybrid model that combines in-person and virtual visits (12). Maternal healthcare providers globally have rapidly adopted telemedicine and virtual care practices for prenatal services, reducing in-person visits and potential exposure to COVID-19 (13). Moreover, specific obstetric telehealth programs were created to monitor pregnant patients with suspected or confirmed COVID-19 (14). These policies helped curb the spread of COVID-19, but several studies have shown that they presented challenges to the provision and experience of maternity care.

Quantitative studies exploring parental experiences reported reduced accessibility, decreased utilization, and lower perceived quality of maternal health services due to lack of technological access and literacy, reduced mobility, and fear of infection, but also distress and confusion during antenatal care associated with (frequently changing) COVID-19-related procedures (15–18). These studies also showed inadequate intra-partum pain relief, increased medicalization of birth, and reduced post-partum skin-to-skin contact and mother-infant bonding. Moreover, they showed that the importance of support persons was undervalued (10, 11, 19) and an increased likelihood of maternal depression and anxiety (20). A mixed-methods study exploring the ability of health workers to provide respectful maternity care during the pandemic showed that the preventive measures led to less family involvement, reduced emotional and physical support for women, compromised standards of care, increased exposure to medically unjustified caesarean sections, and staff overwhelmed by rapidly changing prevention measures (21).

Several quantitative studies have specifically explored whether and how the COVID-19 pandemic exacerbated existing disparities in maternal health outcomes for migrant women and racialized women. A study in the UK showed that pregnant women hospitalized with COVID-19 were more likely to belong to an ethnic minority (22). A multi-center study in Europe showed no difference in the overall perception of quality of maternal and newborn care around the time of childbirth among migrant and non-immigrant women, although migrant women experienced significantly more difficulties in attending routine antenatal visits, and more barriers in accessing facilities (23). An Italian study showed no differences in prenatal course attendance between Italian and immigrant women, yet found that immigrant women would attend them more frequently in hospital settings, instead of in non-hospital settings or the online version of the course (24) A cohort study in Chile demonstrated that immigrant mothers exhibited a higher proportion of c-sections during the pandemic (25).

Qualitative research in the U.S. showed that health service providers of pregnant migrant women reported diminished access and quality of care, amongst others, due to decreased language accessibility, worsened by pandemic-related restrictions on interpreters. They mention that many pregnant migrants delayed or missed appointments due to confusion about their legal rights and health insurance status, or hesitancy linked to their documentation status (26). Improved telehealth availability was often nullified by the lack of a reliable Internet connection or the lack of appropriate medical interpretation (27). Travel restrictions disrupted traditional postpartum support systems, leading to increased social isolation, loneliness, and stress for migrant mothers (26, 28). Job losses in the informal sectors, due to COVID-19-related restrictions, also left many undocumented migrant families financially unstable, limiting their ability to afford healthcare services during pregnancy (27). A qualitative study on perinatal mental health in Belgium showed that pregnant migrant women experienced heightened stress and isolation due to COVID-19 restrictions, which limited antenatal follow-up and heightened risks of postpartum depression and anxiety (29).

Many studies have used a public health framework to understand the impact of COVID-19 on maternal health outcomes of minority populations, primarily focusing on barriers to healthcare access and using the WHO framework of Quality Maternal and Newborn Care as a guiding lens. Most qualitative studies also relied on data provided by the healthcare providers and focused on migration rather than race as an interpretive lens. This study wants to enrich the existing literature by taking the lived experiences of women of color as the starting point and by studying them through the lens of Critical Race Theory, which will be discussed in the following section. Specifically, this study explores how COVID-19 and the policies to contain it influenced the lived experiences with maternity care of Black Sub-Saharan African (SSA) migrants in Belgium.

## Theoretical framework: Critical Race Theory

CRT enables the researcher to examine how structural racism and intersecting forms of discrimination influence inequities in care. Critical Race Theory originated in U.S. legal scholarship during the 1970s, emerging as a response to the limitations of civil rights legislation in dismantling systemic racism (30). It has since been adapted across disciplines, including education, sociology, and increasingly public health. In this context, CRT provides a framework for interrogating how systemic and institutional racism affects health outcomes, particularly among racialized and marginalized populations (31).

CRT challenges dominant ideologies that frame health disparities as primarily resulting from cultural differences or individual choices. Instead, it emphasizes the role of structural forces, including immigration policy, implicit racial bias in healthcare, and socioeconomic inequality, in shaping unequal health outcomes (32). Applying CRT to public health shifts the analytical lens from individual-level explanations to a critical examination of how power and privilege are distributed across racial lines within healthcare systems.

According to CRT, racism is a prevalent and persistent aspect of society rather than an irrational, individual phenomenon. In the healthcare context, this means that discriminatory practices are often embedded in routine procedures and institutional policies, even when they are not explicitly racist (33). CRT allows for a critical analysis of how barriers in healthcare access are not simply the result of bureaucratic inefficiencies but of systemic exclusion rooted in racialized and colonial histories. Also, CRT recognizes that individuals do not experience racism, sexism, or classism in isolation, but rather as interconnected systems of oppression.

CRT prioritizes the lived experiences and narratives of marginalized groups as valid and necessary forms of knowledge (34). These experiences serve as counter-narratives challenging dominant health discourses - in this context, specifically the policies to contain COVID-19 - that often overlook the specific needs of people of color. CRT posits that legal reforms benefiting racialized minorities often only occur when they align with the interests of the dominant group (35). It prompts critical reflection on whether pandemic-era health measures, such as telemedicine or visitor restrictions, were designed with marginalized populations in mind or whether their needs were met only incidentally. In this study, the perspectives of the participants are treated not merely as data but as critical insights that reveal the limitations of the Belgian maternity care system, particularly in times of crisis. Black Sub-Saharan African migrant women in Belgium are positioned at the intersection of multiple social identities, each of which shapes their experiences of maternity care (36).

CRT informs not only what questions are asked but also how data are interpreted, emphasizing structural over individual explanations and pushing for policy implications that address the root causes of health inequities. CRT not only helps expose the mechanisms of exclusion but also provides a roadmap for anti-racist and equity-oriented reforms in healthcare policy and practice.

## Methods

### Data collection

This study is a qualitative study combining semi-structured in-depth interviews and ethnographic observation. Thirty Black, Sub-Saharan African women living in different cities in Belgium participated in the study, all of them first-generation migrants, of whom 26 had a face-to-face interview and 4 online. Participants were recruited through snowball sampling, taking the first author's acquaintances, as a Black Sub-Saharan African migrant woman in Belgium, as a starting point. To ensure the variety of the sample, an additional advertisement was made on social media, resulting in the participation of 12 women. The women came from 11 SSA countries: Rwanda, Burundi, Cameroon, Eritrea, Ethiopia, Guinea-Conakry, Somalia, Uganda, Nigeria, Ghana, and the DRC Congo. The ages of the participants ranged from 25 to 46. All women who delivered a baby during the COVID-19 pandemic, of whom 8 delivered for the first time. The interviews happened in English (20) or French (10), languages in which the authors are conversant. Although over half of the participants had a university degree, most women were working in cleaning jobs or were unemployed (see Table 1), reflecting well-known discrimination of women of color in the Belgian job market (37).

**Table 1:** Characteristics of participants.

Characteristics	Variables	Frequency
Language	English	20
	French	10
Age range	25–35	13
	36–46	17
Duration of stay in Belgium	0–1 year	2
	1–5 years	18
	5 years and above	10
Education	High School	13
	Bachelor	10
	Masters	5
	PhD	2
Civil status	Married	12
	Single	8
	Divorced	3
	Cohabitation with a partner	7
Immigration status	Family Reunion	8
	Student	8
	Naturalized	4
	Permanent Resident	6
	Undocumented Resident	4
Number of kids	1	8
	2	10
	3	6
	4	6
Method of delivery	Vaginal birth	12
	Caesarean birth	18
Occupation	Knowledge worker	7
	Manual labour	17
	Unemployed	6

The interview guide was designed to elicit participants' lived experiences and narratives. Initially they were asked to describe how COVID-19 restrictions had affected their pregnancy experience, delivery and postpartum care. Women who had other babies before COVID-19 were asked to compare both experiences. Guided by CRT's emphasis on counter-storytelling, they were then asked if they agreed that the covid-19 related policies, such as telemedicine and visitor bans, were the same, and had the same impact, for all pregnant women in Belgium. The interviewer probed specifically for moments when they felt their race or residence status shaped their communation and interactions with healthcare providers or the hospital during the pandemic.

Participant observation involved the first author's attendance at a *Born house*—a ceremony to name the child and to welcome the mother and child at home, which is a custom in some of the societies where the women originate from and is continued in Belgium after migration. Black SSA migrants in Belgium continue this ritual in their churches as part of baptism ceremonies and in their monthly community gatherings. These gatherings bring women together as they exchange stories about childbirth, parenting, and motherhood. Although COVID-19 restrictions stopped these gatherings between 2019 and 2021, the first author attended four of these gatherings in 2022, when the restrictions were lifted.

The data collected through interviews and participant observation were all transcribed immediately after interacting with the participants. Thematic saturation was assessed iteratively throughout the process of data collection. After every two to three interviews, preliminary codes and themes were compared by the researcher to determine whether additional interviews contributed new information. Guided by Critical Race Theory as an analytic lens, saturation was interpreted not merely as repetition of responses but as the point at which participants' narratives provided sufficient depth to analyze how structural racism, COVID-19 policies, and migrant status intersected.

### Data analysis

After transcription, both authors reviewed the scripts several times, using the qualitative data software, NVivo14. The thematic analysis proceeded in three phases. First, initial open coding was used to capture participants' lived experiences of maternity care during COVID-19 without imposing predefined categories. During this phase, codes were generated inductively, and emerging themes were identified and compared across interviews. Themes that appeared relevant to the study objectives were explored further in subsequent interviews to deepen conceptual understanding.

In the second phase, the analysis turned to passages containing keywords that could be regarded as “counterpoints” (38) meaning, in the context of coding, words, phrases, or concepts articulated perspectives diverging from, or standing in tension with a dominant narrative. This approach aligns with CRT, which emphasizes the critical interrogation of dominant narratives and the centering of counter-stories. Examining counterpoints allowed us to identify moments where universal public health discourses about the covid-19 measures were resisted or reframed, but also to better understand internal heterogeneity within participants' accounts.

In the third phase codes were then organized into higher-order themes. This process was informed by foundational constructs derived from CRT, such as racialization, racism and structural power relations. The findings were then compared to already existing empirical and theoretical literature. The findings were compared to already existing empirical and theoretical literature. Consistent with CRT's commitments, interpretations prioritized structural mechanisms rather than individual-level explanations.

## Study context

Belgium has a universal public healthcare system, although undocumented migrants can only access healthcare after going through a parallel administrative registration procedure under the Law on Urgent Medical Aid (UMA) (Royal Decree of 12/12/1996). Despite this universal access, migrants face significant structural barriers in the Belgian healthcare system. These include language barriers, lack of culturally responsive care, discriminatory treatment within institutions, and increasingly restrictive implementation of the Law on Urgent Medical Aid (39).

The Belgian Federal Health Care Knowledge Center 2019 report states that foreign-born women, especially from Sub-Saharan Africa, have higher rates of unplanned caesarean sections and reduced prenatal screening. Black Sub-Saharan African migrant women represent a growing population in Belgium, through different migration channels such as family reunification, study visa, asylum, and undocumented migration. They face unique and multifaceted challenges, including racial discrimination, and limited knowledge of, and trust in, the healthcare system.

During the first wave of the pandemic, Belgian hospitals implemented strict visitor restrictions and introduced triage systems that affected access to care (40). Many perinatal health services in Belgium were disrupted. Several public perinatal organizations closed consultation offices for weeks. Gynecological visits were reduced, and the first visit was possible only after the first trimester. Midwives could only conduct home visits when necessary, requiring justification from health authorities. This led to a major shift toward teleconsultations (often without clear guidelines), reduced antenatal visits and monitoring, and patient distrust (16). In subsequent waves, the restrictions became progressively less strict, and the quality of care evolved during the pandemic (17).

## Ethical considerations and positionality

All participants were informed about the research objectives before starting the interview, with the assurance that all participants would remain anonymous. They were also informed of their right to decline participation, interrupt, and withdraw from the conversation at any time. Some participants were reluctant to sign documents, especially women with undocumented status, for reasons of fear. In these cases, oral informed consent was given before each interview, which was documented by the researcher. Some women also did not want to have their interviews audiotaped, in which case, notes were taken and transcribed immediately after the interview. The scripts were stored securely and were only accessible to the authors. Pseudonyms were used to ensure anonymity and confidentiality. The research protocol was reviewed and approved by the medical ethics committee of UZ Brussels (Ethic Committee number: EC-2023-113, BUN: 1432023000099).

In addition to these formal requirements, we paid particular attention to ensuring psychological and emotional safety throughout the interviews. The first author's identity as a mother, a feminist researcher, and a Black, first-generation migrant mother from Cameroon, living in Belgium at the time of the pandemic, facilitated reaching out, creating rapport, and gaining trust from the participants. Several participants actively reached out to make their voices heard. Thereby, we paid special attention to creating a safe space where individuals could express their experiences without fear of repercussion. This was done through setting clear boundaries at the start of the interview, active listening, and anticipating the potential need for follow-up and counselling after participation.

## Results

In this section, we elaborate on four main emerging themes. These themes describe how our participants experienced being pregnant during COVID-19 as (i) a climate of conflicting fears. In this climate, (ii) the interplay between COVID measures and racist assumptions legitimized the dismissal of questions, fear, and pain. They describe how they ended up (iii) consenting to medical interventions without being adequately informed. This left them (iv) feeling wronged, which led them to search for culprits.

### Conflicting fears

Women's narratives revealed the deep emotional tension experienced by Black Sub-Saharan African migrant women as they navigated maternity care in Belgium during the COVID-19 pandemic. Multiple fears overshadowed their pregnancies. Joy, a participant from Ethiopia in a higher-skilled occupation and a single mother, said: “The joy and excitement I thought I would have in having a baby were not there”. The disappointment expressed in the quote reflects a widely shared experience for many women participating in the study. Instead of joy, participants described their pregnancy and delivery as a stressful period, characterized by fear and anxiety. Edit, a married woman from Ghana who was working as a cleaner during the pandemic, said:

Consultations with my doctor and midwife were done over the phone… And it was very stressful to explain all my symptoms. Sometimes, it is after the call ends that I will remember that there are some things I did not tell the doctor. And calling back is stressful because you need to be put on hold, and if the person who picks up does not understand your language, they need to look for a person who can speak your language, and on some days, they will not be able to contact you back, and then the next day, you need to start calling again. I had a lot of anxiety and was afraid that consulting over the phone was not enough.

As described by Marshall et al., the shift to teleconsultations during COVID-19 created additional barriers for pregnant women who did not speak Dutch or French. The limited availability of interpreters, combined with reduced access to maternal care services, forced participants to repeatedly attempt to contact health care providers, generating significant stress. The quote further illustrates how language barrier - together with the process of repeatedly explaining symptoms and waiting on hold - delayed access to timely care, generated feelings of being unheard and heightened fears that pregnancy-related problems would not be detected promptly. Simultaneously, many pregnant women associated medical facilities with danger, as illustrated by Chiamaka, a single mother from Nigeria who was pregnant with a two-year-old:

It was driving me insane to think that I needed to go with my toddler to the hospital for my appointment. I was so stressed that he would get infected that I completely forgot the importance of keeping my doctor's appointment.

In other words, pregnant women navigated a dual concern: minimizing their risk of contracting COVID-19 during hospital visits, while at the same time worrying about the potential impact of limited prenatal care on the health of their unborn child and desiring more comprehensive support. This fear was even higher amongst those women who themselves contracted COVID-19, as illustrated by the following quote from Rosine, a single mother from the Democratic Republic of Congo:

I had hypertension and had also contracted the coronavirus. The doctor asked that I stay overnight at the hospital so I could be monitored. The nurses treated me like they were waiting for me to die. I was abandoned, and I was not checked on. I was afraid for myself and my child, but could not express myself because there was no one to talk to, and when they came to me, they stood very far off, so all I did was just cry and pray (Translated from French).

Rosine's account highlights the extreme vulnerability felt by high-risk pregnant women during the pandemic. She describes abandonment and neglect, as infection-control practices such as staff keeping physical distance combined with a lack of communication, reassurance, and basic care, intensified her fear for both herself and her unborn child. Rather than easing these fears, COVID-19 policies and health-care responses reinforced women's perceptions of being in danger, deepening their anxiety. For women like Rosine, the hospital, supposedly a place of safety, became instead a site of fear and abandonment.

### Intersection COVID-19 policies & race

Participants' narratives did not just reveal mentions that the pandemic was a period of widespread fear, but also how the covid-19 measures reinforced pre-existing racial hierarchies and contributed to a sense of exclusion and discrimination within the healthcare system. Joy, who, as mentioned earlier, regretted that her pregnancy was not a period of joy, adds that her fears were not taken seriously during delivery:

It was as if everywhere you turned, you saw only sad and scary faces with masks. And with my skin color, I feel even more scared and lonely, because I feel I am not understood. I am expected not to be afraid because I am used to it. One nurse suggested that I not need to complain or be scared because they are handling the situation well. After all, where I am coming from, we always have pandemics, and they are not well handled, and she cited the situation of Ebola in Africa. I was so shocked at the insensitivity that I just had to keep to myself till I left the hospital.

Joy describes the fear she felt when surrounded by healthcare workers wearing masks, coupled with heightened self-awareness of her skin color, which intensified her sense of isolation. She also felt unable to voice her fear. This feeling is triggered by a healthcare worker's remark about Joy's race, invoking problematic colonial stereotypes, framing Africa as a continent of disease. The comment additionally assumed that previous life experiences with Ebola outbreaks in Africa would render people less fearful in the context of COVID-19. In Joy's case, this is completely unfounded, considering Joy is from Ethiopia and has never remotely been exposed to such an experience. Moreover, the nurse's remark invokes an assumed superiority in handling medical crises, thereby reproducing another stereotype about an imagined West as a space of medical-technological expertise and superior knowledge, a narrative historically used to legitimize the colonial project. The remark had a tangible impact, causing Joy to stop sharing her feelings with the medical staff. Such comments can lead to important complications, as becomes clear from the story of Vera, a 35-year-old married woman from Eritrea with refugee status. When narrating about her delivery, she mentions:

I had a C-section. My baby was fine. I told the nurses I was not fully healed and wanted to stay back in the hospital for more days, but the nurse said there are not enough beds because of the pandemic, and went ahead and said something referring to African women being strong women and that I can manage the pain. I was very shocked to hear her say that, but I could not imagine that pain was racially incurred. I was feeling pain just like any woman who had a C-section, and the wound was not fully healed. I expected the medical staff to understand and help, but I was told I could manage it because, as an African woman, I am supposed to be strong. I was so shocked and disappointed to the extent that I did not complain about the pain again, even though the pain was bending me over. I went home, and the pain became worse, and my wound burst open. Even though I had already discovered in the hospital that the stitches were opening, I did not complain because of the statement the midwife made. For fear of contracting an infection with an open, unhealed stitch, my husband called the emergency room, and I was rushed back to the hospital. I underwent emergency surgery for the doctor to restitch the open area, and I had to go through the pain all over again.

In the context of limited beds and resources due to COVID-19 – a highly stressful situation- the nurse relied on racial prejudices to decide whether Vera could remain longer in the maternity ward. Vera's postoperative pain and concerns were dismissed, drawing on seemingly innocent stereotypes that portray African women as exceptionally strong or expected to be able to endure pain. The belief that African women are inherently strong and its impact on healthcare provision for Black patients has been documented in the academic literature (41). However, its relevance in shaping the implementation of pandemic policies has not previously been demonstrated. The fact that the nurse voiced this stereotype in a context of bed shortages resonates with research suggesting that heavy workloads and acute stress can reduce healthcare providers' ability to self-monitor and regulate the expressions of implicit bias (42, 43). Vera describes that the disbelief, shock, and disappointment triggered by the nurse's remark had an almost paralyzing effect, making her hesitant to communicate her symptoms or seek further assistance. As her pain worsened, she remained quiet, eventually developing complications that required emergency surgery. A comparable experience was recounted by Pearl, a 28-year-old, married, and highly educated woman from Gabon:

I know in most cases, if you have a successful birth, especially a vaginal birth, you go back to the house after three days. But in my case, I complained that I was still bleeding a lot even though, naturally the bleeding was supposed to have reduced, but I was discharged nonetheless without them addressing the situation, and there was no after-birth follow-up because of the pandemic policy, and they said if there is any complication, I should call the emergency department. They first complained that there were emergency cases that needed beds and care, and my situation was not an emergency. My husband was worried and tried to insist that they should investigate my complaint, but the nurse said she knows most African men do not help their wives at home, but if my husband helps with the house chores and allows me to rest, I will be fine. My husband was so angry and asked that I be discharged immediately. I went home, and two days after, I woke up and my bed was soaked with blood. I was bleeding nonstop, and no amount of sanitary towels could help. I was confused and panicky, my husband called the ambulance, and I was rushed back to the hospital. The doctor had to carry out an emergency surgery on me to stop the bleeding and explained that the bleeding was due to the failure of my uterus to contract.

Pearl experienced continued, heavy bleeding after childbirth. She expressed concerns about the bleeding but was discharged from the hospital with arguments referring to pandemic-related policies and limitations in hospital resources. When her concerns were not taken seriously, her husband advocated for her. Yet instead of being heard, his question was being dismissed by holding him and his role in household chores, responsible for her health, and legitimizing this with stereotypical representations about irresponsible and careless African men. This remark made the couple leave without adequate follow-up and monitoring at home. As argued elsewhere, “showing their heels”, whilst forsaking further care-seeking, allows migrants to invoke feelings of guilt in the healthcare workers. It gives the implicit message to healthcare workers that the remark is inappropriate or discriminatory, and that a better service is desired, yet it can hardly be considered an autonomous decision (44, 45).

### Un-informed consent

Many participants reported making pregnancy-related decisions that were influenced, in whole or in part, by the pandemic context—decisions they later came to regret. Several participants regretted having consented to medical interventions and felt pressured to do so. Elma, a Cameroonian woman who was pregnant for the first time, mentioned:

With me, I think the pandemic measures made my birth experience difficult, because… I believe my experience would have been different if we had not been in lockdown. Firstly, I had to stop work (…). I stayed indoors; thus, I had swollen legs, I could not walk, and I had to call for an emergency and was rushed to the hospital, where it was discovered my blood pressure was very high. I still had two weeks before my due date, but the doctor recommended I have an emergency delivery for fear of further complications if I waited till my due date. I proposed that I should be induced to give birth to the baby, as it is more painful, but I was willing to endure the pain and give birth naturally because I know the recovery process is short. I did not want a C-section because I know I am a single mother with no help. However, the midwife and doctor kept insisting that I might have pre-eclampsia. The procedure would take a longer time, and I would have to spend more time in the hospital, and there were not enough beds, so I had to accept a C-section.

Severe pre-eclampsia can be a valid indication for performing a caesarean section. However, Elma mentions that these are not the arguments that were presented to persuade her. Instead, the midwife and doctor referred to practical concerns such as the hospital's limited capacity, the shortage of beds during COVID-19, and the need to free up space quickly. They emphasized the potential length of an induction and the strain it might place on hospital resources. Elma felt pressured to accept the C-section, not because of a clearly explained medical necessity explained to her, but because of logistical considerations that left her feeling like she had no real choice. This experience made her feel vulnerable and overlooked as a patient, with her preferences and fears sidelined.

The absence of reflectively endorsed consent emerged as a recurrent theme, with participants agreeing to epidurals, the use of assisted delivery tools, C-sections, and early discharge without their genuine consent. This lack of genuine consent aligns with quantitative findings from Galle et al. (17). It was not always the result of direct pressure from the hospital staff, as Chiamaka's experience shows when she tried to express her wish to avoid an epidural during delivery:

When I went for the [antenatal] appointment [where the epidural was discussed], just seeing the way the nurses and doctors were hurrying, I told myself, needless to explain, I will just go with whatever they recommend.

In other words, the pandemic also created an atmosphere where patients empathized with the workload of the healthcare workers, which amplified the power imbalance between patients and healthcare providers, leaving patients feeling discouraged from expressing their preferences. Several preconditions necessary to enable informed consent for our participants were simultaneously under pressure.

Firstly, the COVID-19 measures significantly disrupted communication between healthcare providers and patients, amongst others, by making it impossible to overcome language differences. This is expressed by Sarah, a Senegalese married woman who came to Belgium through family reunification:

During my first two pregnancies, I always went for my maternity care appointments with my husband, and he would help with the interpretation for me to understand better. Because of the COVID rules, no one can come with me; the midwife speaks very fast, and with the mask, I can't see her mouth or her expression (Translated from French).

Sarah speaks Wolof and has limited proficiency in French. Before COVID-19, she managed to communicate with healthcare workers through her husband's mediation. Although some hospitals provide translators and intercultural mediators for certain languages, many patients - like Sarah - rely on informal interpretation by relatives, friends, or even bystanders. COVID-19 measures rendered such informal facilitators impossible. Sarah's experience reveals that the COVID-19 measures did not result in increased efforts to provide professional translators. Instead, communication is described as hastier, and mask-wearing further impeded her understanding of the midwife or the emotional meaning of the information conveyed. One participant reported that these additional language barriers induced by COVID-19 measures intersected with dismissive remarks toward pregnant women who were not fluent in the local language. Chantal, from Rwanda, explained:

I always prefer to communicate in French because that is the language I am comfortable with. When I had my baby, the nurse attending to me did not know I understood Dutch. She was about to end her shift… And the nurse who was supposed to take over after her, I heard her saying to the nurse, that she should pay more attention to the other new mother, who was a Belgian, that I do not understand Dutch, she should not stress herself in trying to explain anything to me. (Translated from French)

The quote illustrates that language differences lead to reduced, instead of the usual or increased, efforts by healthcare providers to provide information to patients, thus triggering and contributing to discrimination in care provision. Moreover, many patients reported feeling burdensome when insisting on establishing effective communication. As Rose mentioned:

In the hospital, one midwife had to go and look for a colleague who could speak French well… At that point, I did not want to be a burden to them anymore, so I just kept saying yes, even though I did not understand. (…) So, I skipped the few appointments that were made and decided to look for information myself. (Translated from French)

Many participants mentioned that the shift to teleconsulting exacerbated language barriers in various ways. When contacting healthcare services, they often faced prolonged wait times. In cases of mutual misunderstanding due to language differences. As noted above, participants were sometimes instructed to leave a phone number for a callback that never occurred. When calls were answered, staff often appeared rushed, and generally no interpretation or meaningful assistance was available. Consequently, participants found themselves caught in a cycle of repeated attempts to initiate contact without resolution. Ultimately, many participants gave up trying to obtain medical advice due to the combined language barriers and experienced discrimination.

Secondly, because of the communication challenges, the pandemic prompted a massive shift toward online and informal gathering of information. While some participants managed to access partial guidance through online antenatal classes, many others disengaged entirely. As a result, several participants did not receive any antenatal care until the later stages of their pregnancy. This is illustrated by the quote of Rose above, but also by the following quote from Cynthia, a Nigerian cleaner and a first-time mother:

I speak English and a little Dutch. During the pandemic, communication barriers were more due to social distancing, and discussions over the telephone were sometimes not clear… I heard about an online prenatal class organized by Kin en Gezin. It was in Dutch, and I could only understand a little. I tried using Google Translate, but it didn't help much. I logged out after 10 min. I felt stupid because everyone else seemed to follow. I just want to know how to care for my baby and what to expect in labor, since it is my first baby. So I had to look for ways to ask other African mothers through WhatsApp, which I believe did not help much, because they were giving me their experiences before the pandemic, and to me, it only creates fear.

Cynthia expresses that her unsuccessful quest for medical information is frustrating and has left her feeling inadequate. Many participants reported similar self-blame for not understanding available information. They often turned to family members and friends for guidance. However, as with many participants, Cynthia expressed that much of this information induced additional fear. Furthermore, the information was not tailored to the realities of giving birth in a pandemic context, which involved new procedures, restricted support during labor, and limited direct communication with healthcare providers. As Joy noticed during the delivery, “it often contradicted the information I had from my mum and what the midwife told me”.

### Feeling wronged. Attributing blame. Without consequence

Our participants recounted numerous experiences of unwanted medical interventions as well as complicated deliveries and postpartum complications. Among the thirty participants, ten women had to be readmitted due to postpartum complications. All women who suffered such complications believed that these situations could have been detected and prevented had they been able to stay in the hospital longer after giving birth or had they received the antenatal and postpartum care they were entitled to. This is harrowingly illustrated by the following quote from Claire:

The doctors and nurses were busier with COVID-19 cases than they were with pregnant women. When I called the hospital for an appointment, I was informed that only critical patients were accepted (…). During labor, I was told my baby had died in my stomach]. I have never felt such pain… With no assistance, I was induced to push out the baby, and it was the most horrifying experience of my life. I keep asking the doctors what happened and how come they missed the fact that my baby would not survive, and I keep getting excuses… Maybe they missed the sign due to the pandemic, that my baby had a defect. I could not fully comprehend everything because I was so angry, hurt, and disappointed. And to make everything worse, I was not expected to receive any form of consolation or any help emotionally and psychologically because of the pandemic measures … And when I asked for one, I was told they expected me to be strong. I did not understand the meaning of “strong”, or is it because I am African, and I am not expected to feel pain? (Translated from French)

Claire lost her baby due to an intrauterine death, an extremely painful experience. However, besides the grief, her dominant emotion is anger - anger at the failed attempts to access antenatal care, at the missed detection of early signs of potential defects, at being alone during delivery, and at the lack of emotional support afterward. The medical staff's explanation that pandemic circumstances were to blame is described by Clementine as an excuse. Moreover, she perceives the advice to be strong as misplaced, since it's informed by prevailing stereotypes of strong African women.

Ten participants also noted that, while they had previously delivered their babies naturally before COVID-19, they underwent Caesarean sections during the pandemic. Most expressed concern or disappointment, as illustrated by a quote from Chantal, a woman from Rwanda, who had a C-section due to fibroids that were only discovered during delivery:

I did not know how to tell my mom that I just had a C-section, because I knew how disappointed she would be.

Most women felt these interventions and complications could have been avoided. For Chantal in particular, undergoing a C-section also incurred disapproval from her mother, as women in her lineage are expected to have a virginal birth. Similar to what has been described in Tanzanian research, vaginal birth is perceived by Chantal as a symbol of womanhood and successful motherhood, whereas a C-section is regarded as a sign of failure (46). Feelings of disappointment and anger were frequently accompanied by attempts to identify who or what was to blame. Sometimes, blame was directed toward individual healthcare professionals. Sarah, for instance, held her doctor responsible, stating:

The doctor insisted and gave me a time frame that if I am not fully dilated by then, he will carry out a C-section on me.

Others, like Chantal, attributed responsibility to the local gynecological team, arguing that the C-section was “their fault because of the limited gynecological visits”. Still others, such as Joy, placed blame more broadly on the government, criticizing the measures implemented during the pandemic:

Pandemic measures affected my birth experience, and if I were given an option and I could see the future, I would not have been pregnant during the pandemic.

Lastly, some respondents felt left alone by their trusted healthcare professionals. As Karen from Ghana expressed:

It was frustrating because a few weeks ago, the social workers were very receptive, but now they are no longer willing to help

Problems with healthcare access were, in themselves, not new to many of the respondents. Florence, an unemployed single mother from Senegal who came to Belgium on a student visa and later lost her residence permit, reflected on her second pregnancy during the pandemic:

The pandemic made things more difficult for me. The measures affected my childbirth experience… I depend a lot on the community and social support because I do not have the right papers to live in Belgium. I usually receive food, material, and emotional assistance from the PSWO [Public Social Welfare Office]. They were closed during the pandemic, and things were difficult. I depended on them to assist with my application for medical coverage. The pandemic made it difficult to have access to this support, especially medical assistance. I did not know how I would pay for my medical bills. (Translated from French)

Florence's remark that “things got more difficult” implicitly points to pre-existing difficulties. Before the pandemic, she regularly interacted with the public social welfare office responsible for the implementation of the Urgent Medical Aid (UMA) Act, the legal framework regulating healthcare access of undocumented women. Several studies have shown that undocumented migrants often rely on informal networking strategies to overcome the barriers associated with UMA procedures. Florence, for instance, received support from friends, peer networks, social workers, lawyers, and church volunteers. In addition to providing material and emotional support, these actors helped her navigate the burdensome administrative process required to access healthcare. Many participants expressed a preference for local community health centers for their antenatal care as their services are tailored to patients' specific needs and often extend beyond just clinical support. However, as illustrated by Karen, during the pandemic, it became increasingly difficult to rely on these centers and trusted healthcare workers, since they were either closed or less accessible. This highlights that COVID-19 measures not only reduced access to the public welfare services and first-line maternity care services but also undermined usual support systems and informal coping strategies. Restrictions on mobility and social gatherings further limited efforts to navigate the healthcare system, particularly for undocumented migrant women and those with a precarious residence status.

Although many participants expressed sadness or anger about the way they were treated or abandoned, none reported pursuing formal complaint procedures, legal action, or repair. Although blame was being assigned, it remained without consequence or redress.

## Discussion

The COVID-19 pandemic generated pervasive fear among all pregnant Black migrant women we interviewed. On the one hand, fear of contracting the virus and the restrictions imposed led some women to avoid seeking antenatal care. Simultaneously, many feared for the health of their unborn child due to being denied necessary antenatal or maternity services. While such fears were probably shared by many other pregnant women, our data show that for Black migrant women in Belgium, they were compounded and exacerbated by experiences of stereotyping and institutional neglect as well as linguistic racism and obstetric violence.

Our participants experienced that their pain - both physical and emotional - was frequently ignored or minimized during labour, delivery, and postpartum care. Our data demonstrate the impact of the myth of the “strong black woman”, a seemingly harmless stereotype that contributes to the systematic under-treatment of pain among Black patients (41, 47). This stereotype simultaneously renders black migrant women hyper-visible as racialized others presumed to be immune to a disease affecting an imaginary us, while making them invisible as patients deserving of care, empathy, and protection. Our data show that such stereotyping contributed to inadequate pain management, delayed diagnoses, and a lack of emotional support. The frequency of these dynamics in the context of the COVID-19 crisis corroborates existing research indicating that time pressure, high emotional pressure, stress, and fatigue reduce healthcare professionals' ability to self-monitor and regulate the expression of implicit racial bias (43, 48).

Our data also demonstrate that the measures implemented to contain COVID-19 – specifically mask-wearing, teleconsultations, and the reduced availability of interpreters – exacerbated existing language barriers in interactions with maternal health providers. While communication challenges related to language differences and limited interpretation services are not unique to the pandemic (49, 50) their impact was intensified by the decreased accessibility of trusted primary healthcare providers and community support networks. Together, these factors contributed to inadequate access to antenatal care for many participants. As a result, many Black migrant women disengaged from seeking antenatal services altogether, leading to gaps in knowledge about perinatal care, particularly regarding possible delivery methods and their indications - as has also been described in other contexts (51). Moreover, this disengagement led to a shift toward seeking information through informal and online sources.

This information did not prepare pregnant women for giving birth in a pandemic context, raising challenges for informed consent. Many participants reported feeling coerced or not being consulted about interventions during delivery – experiences that can be understood as forms of obstetric violence. These findings align with pre-COVID research of Meyer et al., which showed that migrant women in Switzerland were more likely to experience coercion in maternity care. Language barriers, likely intersecting with systemic paternalism and colonial representations about ignorance or submissiveness, made it easier for medical staff to override consent protocols, particularly under the guise of a pandemic urgency (52). As illustrated by Shantal's experience, language differences also sometimes triggered healthcare providers to pay less attention to her needs. This reflects findings by Lipovec Čebron et al., who describe how language barriers in healthcare settings can exacerbate racist attitudes toward migrants and ethnic minorities, phenomena that can be described as linguistic racism (53). Taken together, these elements highlight the intersecting roles of race, language, and migration in shaping experiences of obstetric violence.

After their deliveries, many participants expressed a sense of being wronged and violated by maternal healthcare professionals or systems during the COVID-19 pandemic, contributing to what others have termed *racial trauma* in medicine (54). Racial trauma is often delegitimized or pathologized in dominant narratives. Studying the experiences of our participants through the lens of CRT urges us not to view their anger as irrational. CRT asserts that racism is not an aberration but a permanent and normalized feature of society. It calls for centering of marginalized voices and recognizing the experiential knowledge of people of color as a form of counter-storytelling that challenges dominant narratives of equity in healthcare (53).

CRT further highlights that neutral and supposedly color-blind policies often produce racially disparate impacts. The introduction of public health measures, without consideration for the specific needs of migrants and linguistic minorities, can be considered as a form of institutional neglect that reinforced existing inequities. Interpreted through that lens, COVID-19 measures, though formally race-neutral, were implemented in ways that amplified racist assumptions and enabled caregivers to justify dismissive behaviors, under the guise of clinical necessity (55). The shift toward informal sources of information is not understood as a personal failure, but as a rational response to structural exclusion. From a CRT perspective, recurrent breaches of consent procedures are not considered as isolated incidents but as institutionalized racism where Black migrant women are stripped of agency (56). Moreover, the public health measures also disproportionally affected undocumented migrants, the *racialized other par excellence* (57), by obstructing the implementation of the very policies that are supposed to govern their access to healthcare services.

Maternal healthcare systems are not the sole causal factor contributing to adverse maternal events or health outcomes. These outcomes are deeply intertwined with broader social determinants of health. Moreover, women's lived experiences of maternity care are also shaped by encounters with structural racism in other societal domains, such as the labour market or the housing market, which influence how the quality of care is perceived. Minkoff has argued that the solution to address racial disparities in adverse maternal outcomes during COVID-19 cannot be found within the walls of hospitals (11). Indeed, the pandemic did not create the structural determinants of these disparities. Nevertheless, the well-intended and seemingly neutral measures to contain it created yet another barrier for maternity services of Black migrant women. Their experiences shed light on the intersecting roles of race, gender, language, and migration status in obstetric violence. Therefore, unlike Minkoff, we argue that it is mistaken to view structural racism as a societal process taking place outside the walls of the hospital - something maternity services merely try to remedy. Rather, maternity services unfortunately participate in and reproduce this societal process.

## Strengths and limitations

The use of snowball sampling to recruit participants may have introduced both selection bias and self-selection bias. The social identity of the first author may selectively have facilitated the recruitment of participants with a similar cultural or religious background. Women who have had particularly difficult or traumatic pregnancy experiences might also have been more motivated to participate. In addition, participants asked to suggest new participants may have been inclined to refer individuals with similar experiences and ethnic or religious backgrounds. Nevertheless, this does not invalidate the findings, as the study sought to explore the lived experiences of women during pregnancy and childbirth in the pandemic. Moreover, the researchers actively strived to as much variety as possible in order to select a representative sample. The richness and consistency of the narratives provided valuable insights into systemic gaps in maternity care that might otherwise go unreported.

A particular strength of the study is that it successfully recruited 30 participants from a hard-to-reach, marginalized population, a group that is often underrepresented in research. This was made possible, in part, because the first author, who conducted all the interviews, shared several identity markers and life experiences with the participants. This positionality facilitated the development of rapport and trust, creating a safe and empathetic space in which participants felt comfortable sharing their experiences openly and in depth.

## Conclusion

Documenting how first-generation Black migrant women living in Belgium experienced maternity care during the COVID-19 crisis highlights the complex relationship between addressing structural exclusion and crises. The measures introduced to contain the pandemic exacerbated existing structural racism within maternity care services. These findings underline the importance of the development of antiracist policies within and beyond healthcare institutions as well as the need for targeted interventions that improve maternity care systems for all women. Simultaneously, our study shows that tackling structural racism is not only a long-term project but also a matter of awareness of the specific needs of racialized minorities when introducing new, ostensibly universal public health measures.

## Data Availability

The data supporting the findings of this study are available from the corresponding author upon reasonable request.
